# Selective Electron Beam Patterning of Oxygen‐Doped WSe_2_ for Seamless Lateral Junction Transistors

**DOI:** 10.1002/advs.202202465

**Published:** 2022-07-19

**Authors:** Tien Dat Ngo, Min Sup Choi, Myeongjin Lee, Fida Ali, Yasir Hassan, Nasir Ali, Song Liu, Changgu Lee, James Hone, Won Jong Yoo

**Affiliations:** ^1^ SKKU Advanced Institute of Nano Technology Sungkyunkwan University Suwon Gyeonggi‐do 16419 Korea; ^2^ Department of Mechanical Engineering Columbia University New York NY 10027 USA; ^3^ School of Mechanical Engineering Sungkyunkwan University Suwon Gyeonggi‐do 16419 South Korea

**Keywords:** 2D semiconductors, e‐beam irradiation, oxygen plasma, patterning doping profiles, tungsten oxide

## Abstract

Surface charge transfer doping (SCTD) using oxygen plasma to form a p‐type dopant oxide layer on transition metal dichalcogenide (TMDs) is a promising doping technique for 2D TMDs field‐effect transistors (FETs). However, patternability of SCTD is a key challenge to effectively switch FETs. Herein, a simple method to selectively pattern degenerately p‐type (p^+^)‐doped WSe_2_ FETs via electron beam (e‐beam) irradiation is reported. The effect of the selective e‐beam irradiation is confirmed by the gate‐tunable optical responses of seamless lateral p^+^–p diodes. The OFF state of the devices by inducing trapped charges via selective e‐beam irradiation onto a desired channel area in p^+^‐doped WSe_2_, which is in sharp contrast to globally p^+^‐doped WSe_2_ FETs, is realized. Selective e‐beam irradiation of the PMMA‐passivated p^+^‐WSe_2_ enables accurate control of the threshold voltage (*V*_th_) of WSe_2_ devices by varying the pattern size and e‐beam dose, while preserving the low contact resistance. By utilizing hBN as the gate dielectric, high‐performance WSe_2_ p‐FETs with a saturation current of −280 µA µm^−1^ and on/off ratio of 10^9^ are achieved. This study's technique demonstrates a facile approach to obtain high‐performance TMD p‐FETs by e‐beam irradiation, enabling efficient switching and patternability toward various junction devices.

## Graphical abstract

The authors utilize a selective e‐beam irradiation to pattern the doping profile of oxidized p^+^‐WSe_2_ field‐effect transistors (FETs), overcoming the key challenge of surface charge transfer doping. Seamless lateral junction p‐FETs are realized with high on/off ratio (10^9^) and saturation current (−280 *μ*A µm^−1^), which are crucial for designing logic circuits.

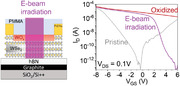

## 1 Introduction

Two‐dimensional (2D) semiconductors, such as transition metal dichalcogenides (TMDs), are promising candidates in modern electronic devices because of their efficient electrostatic tunability owing to their atomic thinness and dangling bond‐free surface. However, the Schottky nature of metal–semiconductor (MS) interfaces, due to Fermi level pinning contributing to the unacceptably high contact resistance (*R*_C_), limits the implementation of 2D TMDs in the silicon complementary metal‐oxide‐semiconductor (CMOS) technology.^[^ ^1^, ^2^, ^3^, ^4^ ^]^ To overcome this barrier and further improve the performance of TMDs field‐effect transistors (FETs), effective doping methods for 2D materials are in high demand. However, ion implantation used in conventional Si technology is not compatible with 2D TMDs owing to the fragile thin body against high‐energy ion bombardment. Various doping methods have been proposed for 2D materials, such as substitutional doping,^[^ ^5^, ^6^ ^]^ intercalation doping^[^ ^7^, ^8^, ^9^ ^]^ and electrostatic doping.^[^ ^10^, ^11^ ^]^ Nevertheless, such strategies have had limited success in terms of long‐term stability, low device‐to‐device variability, and low‐temperature processing.

Surface charge transfer doping (SCTD) has been proposed as an effective alternative for ion implantation in 2D TMDs. SCTD can be realized from the difference in work functions between the host material and dopant, which determines the extent of charge transfer and the type of doping.^[^ ^2^ ^]^ Among the dopants used for SCTD, solid‐state dopants exhibit the advantages of high repeatability, long‐term stability, and CMOS compatibility. Recently, the formation of solid‐state non‐stoichiometric tungsten oxide (WO*_x_*) using oxygen plasma or UV–ozone treatments, which induces a strong p‐doping effect on the underlying TMD layer, has been studied to overcome the drawbacks of TMDs by improving *R*_C_ of the 2D TMDs FETs.^[^ ^12^, ^13^, ^14^, ^15^, ^16^, ^17^, ^18^, ^19^, ^20^, ^21^, ^22^, ^23^ ^]^ Furthermore, protection of the underlying layer by the self‐limiting nature of the oxidation process has been demonstrated.^[^ ^24^ ^]^ However, a lack of area‐selective doping leading to a degenerately doped channel leads to poor gate tunability of the 2D FETs, which is unfavorable for logic devices.^[^ ^12^, ^13^ ^]^ Thus, it is important to develop a facile and patternable doping technique to ensure the OFF state of FETs while maintaining a high doping concentration at the contact regions to reduce *R*_C_. Efforts have been made to accomplish this goal by covering the channel area with either a photoresist or hBN prior to oxidation at the contact area of WSe_2_ FETs.^[^ ^12^, ^13^, ^14^ ^]^ Such contact‐doped WSe_2_ FETs with seamless junctions exhibit significantly improved on/off ratio, mobility, and *R*_C_. Although high‐performance FETs have been obtained with such techniques, the contact area can be exposed to the ambient atmosphere, leading to device instability. Moreover, the mechanical transfer of hBN flakes to protect the channel region against oxidation hinders the precise positioning of the protecting layer and the formation of short channels for further scaled devices.

In this study, we present a facile method to accurately pattern the SCTD profiles of degenerate p‐type (p^+^)‐doped WSe_2_ FETs using electron beam lithography (EBL). Low‐energy e‐beam exposure n‐dopes TMDs by generating an electrostatic built‐in potential through the formation of trapped charges in the dielectric layer.^[^ ^18^, ^19^, ^20^, ^21^, ^22^ ^]^ The n‐type doping effect on p^+^‐WSe_2_ was confirmed by optical measurements and gate‐tunable photovoltaic effect of a lateral p^+^–p seamless junction diode. Thus, a weakly p‐doped region with significantly enhanced gate tunability was formed by selectively irradiating an e‐beam onto a desired channel area within O_2_ plasma doped WSe_2_, resulting in a high on/off ratio. Owing to the capability of precise patterning via EBL, *V*_th_ of WSe_2_ FETs with e‐beam patterns was accurately modulated by varying the pattern lengths and e‐beam doses while preserving its low *R*_C_. By utilizing thin hBN as a gate dielectric and graphite as a gate electrode with our technique, high‐performance WSe_2_ p‐FETs by sub‐100 nm e‐beam patterning were demonstrated with a saturation current of −280 µA µm^−1^, on/off ratio of 10^9^, room temperature mobility of 108 cm^2^ V^−1^ s^−1^ and low *R*_C_ of 4 kΩ µm. Therefore, our technique provides a simple and facile approach with efficient switching and selective patterning for short‐channel and seamless lateral junction 2D devices.

## 2 Results and Discussion

**Figure 1:**
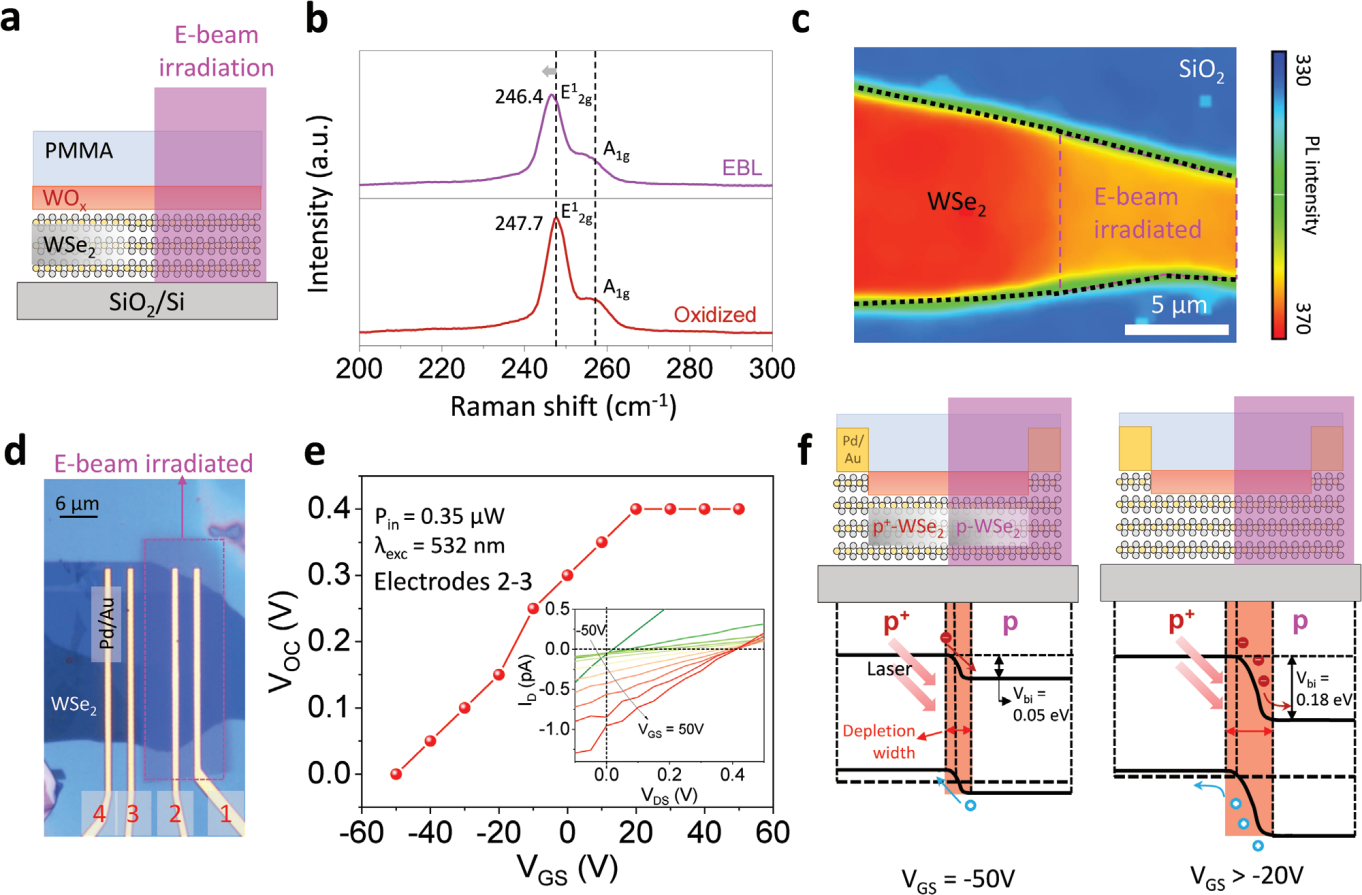
Oxidized WSe_2_ with e‐beam irradiation. a) Schematic of oxidized WSe_2_ sample used for optical measurements to probe the doping effect of e‐beam irradiation. b) Raman spectra of the oxidized WSe_2_ before and after e‐beam irradiation. c) PL intensity mapping at 760 nm (≈1.64 eV) displays a neutral exciton of e‐beam irradiated and non‐irradiated oxidized WSe_2_. d) Optical image of seamless junction diode made of oxidized WSe_2_ patterned by e‐beam irradiation. e) *V*_OC_ at various *V*_GS_ measured from the seamless junction diode (measured between electrodes 2 and 3). The inset shows output curves at different *V*_GS_ under laser illumination with a power of 0.35 µW, wherein the curves indicate the *V*_OC_ modulation in photovoltaic effect. f) Band alignment of the p^+^–p WSe_2_ homojunction at *V*_GS_ = (−50 and −20) V.

Patterning the SCTD profile of p^+^‐doped WSe_2_ FETs is critical for further implementing this doping method for high‐performance switching devices. Our group previously demonstrated the polarity control of p^+^‐type MoTe_2_ FETs using e‐beam irradiation,^[^ ^18^ ^]^ prompting us to employ e‐beam irradiation to realize high‐performance oxidized WSe_2_ FETs with high on/off ratios and short channel lengths by patterning the doping concentration. To probe the doping effect of the e‐beam irradiation, optical measurements of oxidized p^+^‐WSe_2_ were conducted using Raman and photoluminescence (PL) spectroscopy. The WSe_2_ flakes were oxidized to form a WO*_x_* layer prior to PMMA passivation. Standard EBL was performed on a part of the flake to simultaneously examine the exposed and unexposed areas (**Figure** **1**a). The detailed process is described in the Experimental Section. Figure 1b shows the Raman spectra of oxidized WSe_2_ before and after e‐beam irradiation. The E^1^_2g_ band, corresponding to the in‐plane vibration mode, demonstrated a distinct red shift to lower wavenumbers after e‐beam irradiation. Similarly, the position of A_1g_ representing the out‐of‐plane vibration mode revealed a slight red shift. The shifted Raman peaks, A_1g_ and E^1^_2g_ peaks, induced by e‐beam irradiation, were consistent with a previous study of the Raman shift of n‐type doped WSe_2_ by increasing electron–phonon scattering.^[^ ^25^, ^26^ ^]^ Figure 1c shows the PL intensity mapping results of a neutral exciton (≈1.64 eV) on the e‐beam‐irradiated and non‐irradiated areas of the WSe_2_ flake. The irradiated area distinguishably revealed a uniform and weak PL intensity compared with the non‐irradiated area. The weakened PL intensity originated from the increased number of negative trions, which suppressed the generation of neutral exciton peaks as a result of electron injection due to n‐type doping.^[^ ^26^, ^27^ ^]^ Thus, the optical measurements support the selective formation of a weakly p‐doped area by e‐beam irradiation within the oxidized p^+^‐WSe_2_.

We further investigated the n‐type doping effect of e‐beam irradiation on p^+^‐WSe_2_ using photoresponse measurements. We utilized a structure similar to that used for the PL measurement by fabricating seamless lateral junction diodes made of oxidized WSe_2_ patterned by e‐beam irradiation. Optical image in Figure 1d shows that half the p^+^‐WSe_2_ channel was exposed to the e‐beam after oxidation and PMMA coating to form the p^+^–p junction. Here, an e‐beam irradiation with 10 times loop and 600 µC cm^−2^ area dose was used to enhance the built‐in potential at the junction. Figure S1a, Supporting Information, shows the schematic of the device. We explored the optoelectronic properties of our p^+^–p junction device from the transfer and output characteristics obtained under dark conditions and laser illumination with a wavelength of 532 nm (*λ*_exc_) at various powers (Figure S1b,c, Supporting Information). With the increase in laser power, the photocurrent (*I*_ph_) and photoresponsivity of our junction diode increased monotonically, as shown in Figure S1d, Supporting Information, suggesting the successful formation of the junction.

Furthermore, to confirm the effective charge separation at the junction owing to the built‐in potential formed by e‐beam irradiation, we investigated the photovoltaic effect of our diode, as shown in Figure 1e. The inset of Figure 1e shows the output characteristics of the junction device measured under laser illumination with several gate biases in the range of −50 to 50 V. Under illumination, the device exhibited a shift in the open‐circuit voltage (*V*_OC_) values from 50 to 400 mV as a function of gate bias. The shift in *V*_OC_ can be attributed to the gate‐modulated built‐in potential, which corresponds to the change in the depletion width at the junction. The built‐in potential of a conventional p–n homojunction is generally unchangeable with a global back‐gated device structure. Such a change is typically observed when the charge density is asymmetric at the junction, for example, p^+^–p junction, as one side is strongly gate‐tunable, whereas the other side is less gate‐tunable.^[^ ^28^, ^29^ ^]^ The asymmetric gate tunability of each side results in modulation of the built‐in potential. Figure S1e, Supporting Information, shows the transfer curves of the e‐beam exposed and unexposed areas of our junction diode. Unexposed p^+^‐WSe_2_ showed weak gate tunability, whereas exposed p‐WSe_2_ exhibited strong gate tunability, which contributed to the gate‐modulated built‐in potential.

The band diagrams of our p^+^–p junction diode at different gate biases, as illustrated in Figure 1f, quantitatively explain the gate‐tunable *V*_OC_. The Fermi level shifts of both the p‐ and p^+^‐sides at the junction under different gate biases were calculated using the equation $n\;=n_{i}\;e^{(E_{F}\;-\;E_{i})/kT}$, where *n* is the carrier density of the device, *n*_i_ is the intrinsic carrier density, *kT* is the thermal energy, and *E*_F_(*E*_i_) is the Fermi level (intrinsic Fermi level). The carrier densities were extracted from the transfer curves, as shown in Figure S1e and Table S1, Supporting Information. At *V*_GS_ = −50 V, the work functions of p^+^‐WSe_2_ and p‐WSe_2_ were calculated as 5.03 and 4.98 eV, respectively. Thus, the built‐in potential at *V*_GS_ = −50 V was expected to be 0.05 eV. Meanwhile, at *V*_GS_ = −20 V, the work functions of p^+^‐WSe_2_ and p‐WSe_2_ were calculated to be 5.02 and 4.84 eV, respectively, contributing to an increase in built‐in potential to 0.18 eV. Note that the change in the work function of p^+^‐WSe_2_ at different gate biases was negligible compared to that of p‐WSe_2_, because the high hole concentration accumulated in p^+^‐WSe_2_ led to less effective gate control of the carrier density. The change in the built‐in potential of 0.05–0.18 eV was well matched with our observation of the gate‐tunable *V*_OC_ of 0–0.15 V. Thus, the modulation of Fermi level of the seamless junction diode can be quantitatively examined from the photoresponse measurements, corroborating our observation of the n‐type doping effect on p^+^‐WSe_2_ by e‐beam irradiation. Furthermore, we measured the photoresponse of p^+^‐WSe_2_ FETs without e‐beam irradiation as a control device, demonstrating negligible changes in the transfer curves under illumination (Figure S1f, Supporting Information). This further confirms that the n‐doping effect of e‐beam irradiation on p^+^‐WSe_2_ causes the formation of a seamless p^+^–p lateral junction. This observation motivated the formation of a p^+^–p–p^+^ seamless junction to overcome the drawbacks of the SCTD technique for high‐performance short‐channel switching devices, as discussed below.

**Figure 2:**
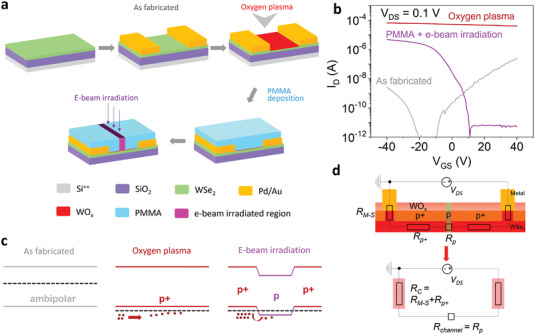
Electrical analysis of the device according to fabrication steps. a) Schematic of device fabrication, b) transfer curves, and c) band structures of the device at different process steps; as‐fabricated, oxygen plasma treated, and e‐beam irradiated after PMMA passivation. d) Equivalent circuit of the device consisting of three resistances in series (*R*_M−S_, *R*_p+_, and *R*_p_). Here, *R*_C_ is expressed as the sum of *R*_M−S_ and *R*_p+_, and *R*_channel_ as *R*_p_.

Next, to overcome the drawbacks of the SCTD method, we utilized the n‐doping effect of e‐beam irradiation by fabricating seamless lateral p^+^–p–p^+^ junction transistors. **Figure** **2**a illustrates the fabrication process of the device, starting from the exfoliation of WSe_2_ on a heavily doped Si substrate covered by 285 nm thick SiO_2_ as a dielectric layer. Few‐layer WSe_2_ flakes (less than 5 nm) were identified by optical contrast. The accurate thickness of the flakes was further confirmed using atomic force microscopy (AFM) (Figure S2a, Supporting Information). The electrodes were patterned by EBL, followed by e‐beam deposition of Pd/Au (5/50 nm) to form MS contacts. Subsequently, the device was treated with oxygen plasma to form a thin WO*_x_* layer to heavily p‐dope the underlying WSe_2_. After oxidation, the device was passivated with 80 nm poly(methyl methacrylate) (PMMA A2 950). Finally, short channels at the middle of p^+^‐WSe_2_ (actual lengths of 70, 120, 500, 1000 and 3000 nm were confirmed by AFM, as shown in Figure S2b and Table S2, Supporting Information) were formed by EBL to render a lateral p^+^–p–p^+^ junction.

Figure 2b,c represents the *I*_D_–*V*_GS_ characteristics at *V*_DS_ = 0.1 V and the corresponding band structures for the fabricated, oxidized, and e‐beam irradiated states. The pristine WSe_2_ FET (grey line) revealed an n‐type dominated ambipolar behavior with relatively high resistance, which represents that the location of the Fermi level of WSe_2_ is near the mid‐gap. For improving the device resistance, we utilized oxygen plasma to form an oxide layer on top of the device. After the oxidation process, degenerate p‐type behavior was produced (red line), showing four orders of magnitude increase in the ON‐current of hole conduction from ≈10^−8^ to ≈10^−4^ A. Therefore, the Fermi level of WSe_2_ was located deep inside the valence band. However, the carrier‐type inversion into p^+^‐type failed to switch off the device, which is unfavorable for designing logic circuits. Thus, it is crucial to maintain or restore the OFF state of the p^+^‐WSe_2_ FETs after the oxidation process, while maintaining a high doping density at the contact area to reduce *R*_C_.

Arnold et al. found that a p‐i‐p homogeneous junction was obtained by covering the middle part of a device with a photoresist before the oxidation process, which improved the electrical performance without vanishing the OFF state of the device.^[^ ^12^ ^]^ Nevertheless, the stability of the device was strongly affected owing to the exposure of the contact area to the environment. Liu et al. utilized a similar device architecture by transferring an hBN flake onto the middle of the channel, which could make it difficult to achieve precise spatial control, as well as short channels for highly integrated device circuits.^[^ ^14^ ^]^ Hence, we employed n‐type doping by e‐beam irradiation on PMMA‐covered p^+^‐WSe_2_ to form a potential barrier by creating a p^+^–p–p^+^ homogenous junction, as illustrated in Figure 2c. We intentionally passivated the p^+^‐WSe_2_ device with PMMA prior to creating a short pattern in the middle part to improve air stability. The transfer curve of the device after e‐beam irradiation (pink line) exhibited unipolar p‐type behavior with a high on/off ratio (>10^6^) with a slightly decreased on‐state current. To figure out which step dominantly reduced the on‐state current in our devices, we measured the electrical characteristics at each process step as shown in Figure S3, Supporting Information. The current (*R*_4pp_) was reduced (increased) dominantly after PMMA coating rather than after e‐beam irradiation. The slightly increased *R*_4pp_ after e‐beam irradiation is presumably due to the induced defects by e‐beam irradiation or a thinned channel by the oxidation process which can enlarge the bandgap.^[^ ^30^, ^31^ ^]^ Nevertheless, we believe that our e‐beam irradiation is still very beneficial to realize an efficient switching behavior as it improved the current on/off ratio significantly.

We further confirmed the local effect of e‐beam irradiation by preparing a control device without e‐beam irradiation (Figure S4, Supporting Information). In sharp contrast with e‐beam‐irradiated devices, the non‐irradiated device exhibited consistent p^+^‐type behavior with a small on/off ratio. Time‐dependent measurements were conducted to investigate the air stability of the proposed technique. As shown in Figure S5, Supporting Information, our PMMA‐covered device displayed almost identical performance, further confirming the device stability after a week. In comparison, the PMMA‐developed device after EBL showed significant degradation after a week, as the surface of the developed region was exposed to the ambient environment. Thus, based on the band structure of the device after e‐beam irradiation as shown in Figure 2c, the e‐beam‐irradiated area is effectively gate‐tunable, whereas the non‐irradiated area preserves its highly p‐doped state. Therefore, the total resistance (*R*_total_) is equivalent to three series resistances: the contact resistance between the metal and semiconductor (2*R*_M–S_), the resistance of p^+^‐WSe_2_ (2*R*_p+_), and the channel resistance (*R*_p_). Here, the e‐beam irradiated area was the effective channel (*R*_channel_ = *R*_p_) of our device, while the remaining part comprising the MS contact and unexposed p^+^‐WSe_2_ was the contact part (*R*_C_ = *R*_M–S_ + *R*_p+_) to further extract the device parameters (Figure 2d).

**Figure 3:**
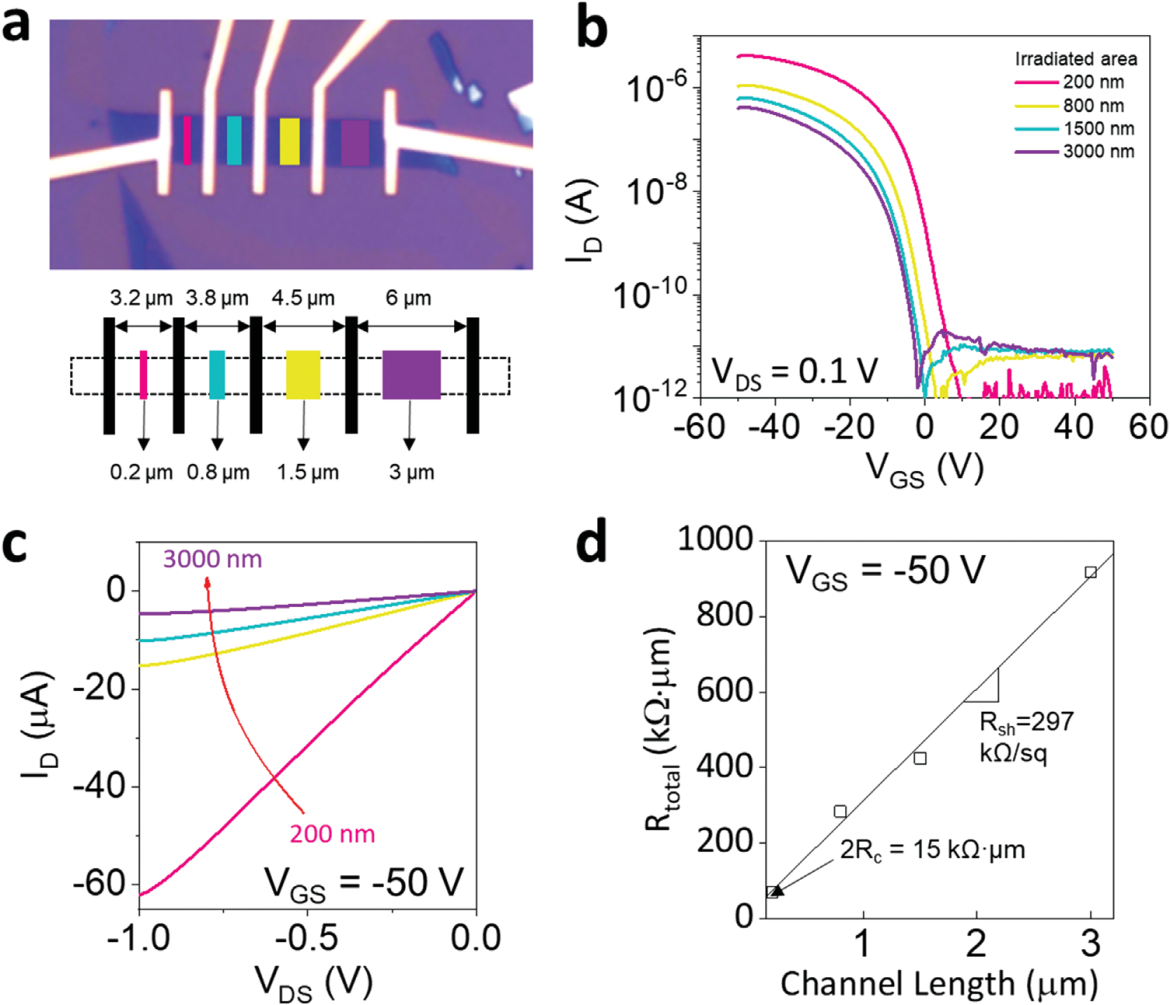
Contact properties of the p^+^–p–p^+^ junction device obtained by TLM measurement. a) Optical micrograph and schematic of the TLM device. b) Transfer curves and c) output curves of devices with several e‐beam irradiated lengths ranging from 200 to 3000 nm with a fixed contact extension length of 1.5 µm. d) *R*_total_ as a function of channel length to extract *R*_C_ and *R*_sh_ by TLM fitting.

Based on the aforementioned device configuration, we quantified the total *R*_C_ of our device using transmission line method (TLM). We fabricated TLM‐structured devices by varying the irradiation length, as shown in **Figure** **3**a. Figure 3b shows the transfer curves of devices with different channel lengths, in which a decrease in the ON current and a negative *V*_th_ shift are observed as the channel length increases. After e‐beam irradiation, a good contact behavior at *V*_GS_ = −50 V was still preserved, regardless of the channel length, as shown in Figure 3c. The output curves of the pristine, oxidized, and e‐beam irradiated devices are demonstrated in Figure S6, Supporting Information. *R*_total_ was extracted from the slope of the output curve. Figure 3d plots *R*_total_ as a function of channel length, demonstrating an excellent linear fit to the data. This indicates the high reliability of our devices owing to the uniform oxidation process and PMMA encapsulation. The *y*‐intercept of the linear fit yielded an *R*_C_ of 7.5 kΩ µm at *V*_GS_ = −50 V, as shown in Figure 3d. The sheet resistance extracted from the slope of the curve was *R*_sh_ = 297 kΩ/sq.

The thickness of PMMA used for passivation also affected the device performance under e‐beam irradiation. Figure S7, Supporting Information, shows the electrical performances of the devices passivated by PMMA A2 950 (thin, ≈80 nm) and PMMA A6 950 (thick, ≈380 nm) (both devices were coated under the same conditions). Note that the A2 and A6 devices have different polymer concentrations, resulting in different thicknesses under the same coating conditions. The extracted *R*_C_ of the A6 device (81 kΩ µm at *V*_GS_ = −50 V) was higher than that of the A2 device (7.5 kΩ µm at *V*_GS_ = −50 V). The higher contact resistance can be attributed to the nonlinearity of the curve, which is likely due to the inaccurate e‐beam patterned lengths written on PMMA A6 caused by the electron scattering in the thick photoresist layers. In contrast, the thin polymer layer with PMMA A2 presented an almost perfectly linear plot. Thus, it was essential to use a thin layer of PMMA to obtain accurate e‐beam‐patterned lengths for this study. To further confirm the effect of the PMMA thickness on our TLM fitting, we performed Monte Carlo simulations to investigate the distribution of scattered electrons within the PMMA A2 and A6 layers. Note that we used the same molecular weight (950 000 g mol^−1^) for both the polymers. As shown in Figure S8, Supporting Information, with a thin layer of PMMA A2, the length of the actual patterns at the WO*_x_*/WSe_2_ layers was close to our intended length from the e‐beam patterns, with a slight difference (≈20 nm) from the length less than 100 nm. The actual length was the same as the e‐beam pattern length when it was >500 nm. In contrast, with thicker PMMA A6, the actual patterned lengths showed significant discrepancies from our intended pattern lengths because of the significantly increased electron scattering, resulting in a wide spatial distribution of scattered electrons within the PMMA layer (the actual length of the e‐beam pattern confirmed by AFM in Figure S2b,c and Table S2, Supporting Information). Thus, we used PMMA A2 for the following devices.

**Figure 4:**
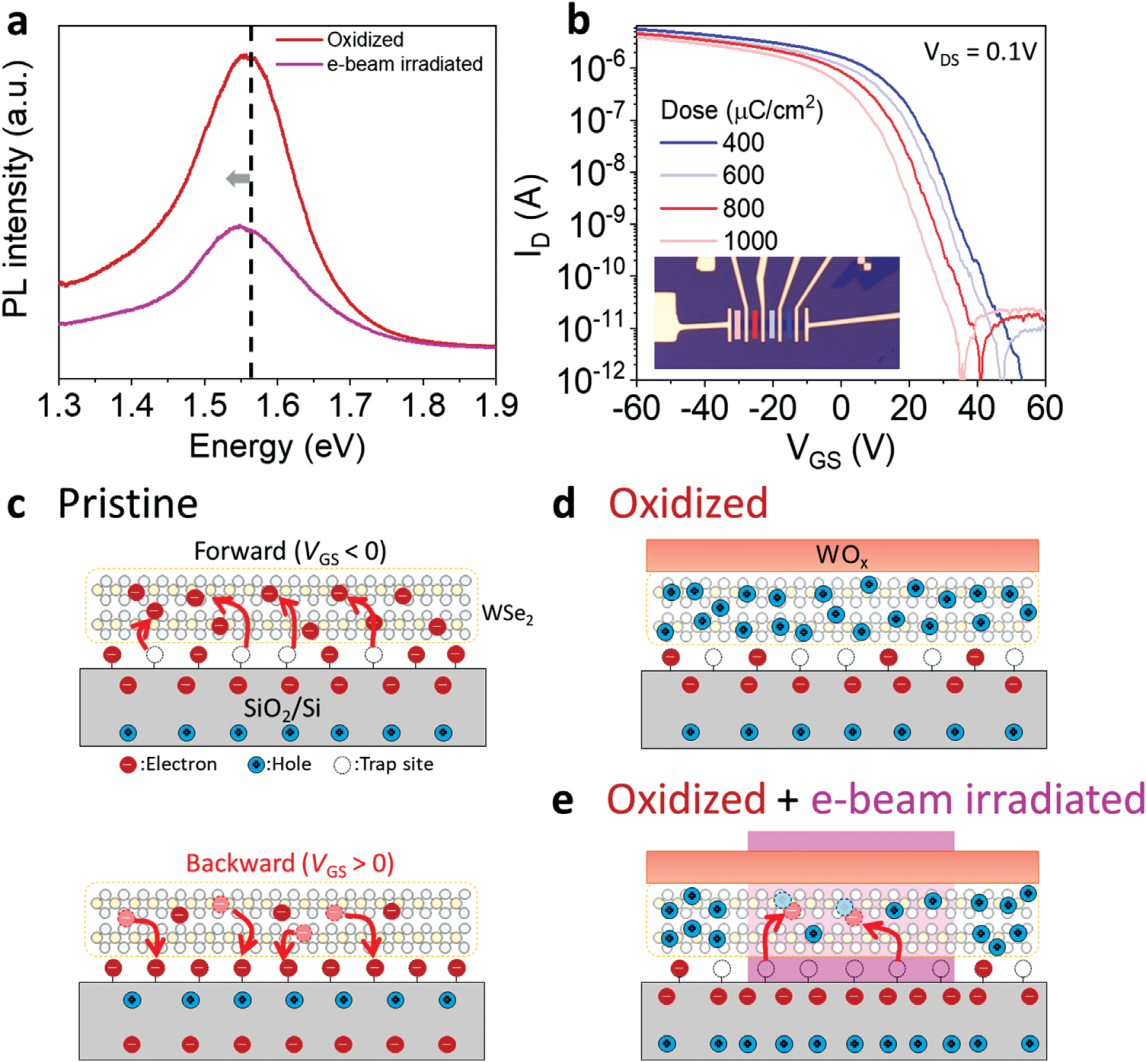
a) PL spectra of WSe_2_ after oxidation and e‐beam irradiation showing a red‐shift in neutral exciton. b) Transfer curves of the devices showing strong dependence on e‐beam area dose ranging from 400 to 1000 µC cm^–2^. The e‐beam irradiated length was fixed at 120 nm for all the tested devices, as shown in the inset. c) Induced hysteresis by interface trap charges at forward and backward bias sweeping for the pristine WSe_2_ device. Charge states in d) oxidized WSe_2_ and e) seamless p^+^–p–p^+^ junction formed by e‐beam irradiation with forward bias considering the interface trap charges as depicted in (c).

To thoroughly understand the effect of e‐beam irradiation, the origin of the reduced doping density of p^+^‐WSe_2_ in the e‐beam‐irradiated area must be determined. This behavior can be explained by several mechanisms, from among which the phase transition in 2D materials can be detected by Raman spectroscopy.^[^ ^32^ ^]^ Based on our results, typical vibration modes of 2H WSe_2_, such as E^1^_2g_ and A_1g_, without any other additional peaks, were observed before and after e‐beam irradiation, as shown in Figure 1b, suggesting that the doping mechanism is not a phase transition. Defects, such as Se vacancies, can induce n‐type doping.^[^ ^33^, ^34^ ^]^ However, the formation of Se vacancies requires a high‐energy process, for example, Ar^+^ plasma treatment or strong e‐beam irradiation with an ultrahigh dose (>4000 µC cm^–2^) typically used in transmission electron microscopy (TEM). Our e‐beam irradiation used the standard EBL process with a low area dose (≈600 µC cm^–2^).^[^ ^33^, ^34^, ^35^ ^]^ Matsunaga et al. found that a low‐energy e‐beam created an S vacancy‐induced strain in the MoS_2_ crystal, which modified the bandgap of MoS_2_.^[^ ^36^ ^]^ The modulation of the band gap caused a blue shift of the neutral excitons after e‐beam irradiation. **Figure** **4**a shows the PL spectra of our p^+^‐WSe_2_ before and after irradiation, demonstrating a red‐shift of the neutral excitons of WSe_2_ before and after e‐beam irradiation, unlike previous report on the strain effect. Such red‐shift indicates the n‐doping effect of p^+^‐WSe_2_.^[^ ^26^ ^]^ Moreover, stronger red‐shift is observed as the e‐beam dose increases (Figure S9, Supporting Information). Thus, defects are unlikely to be formed in the exposed area using the proposed e‐beam irradiation alone (further confirmed in Figure S10, Supporting Information, for the multilayer WSe_2_).

To explore the mechanism responsible for the n‐type doping effect of e‐beam irradiation, we further measured the area dose‐dependent electrical performance, as shown in Figure 4b. Fixed patterns of 120 nm were written by EBL with various beam area doses ranging from 400 to 1000 µC cm^−2^ on an array of p^+^‐WSe_2_ FETs with the same channel width (3 µm) and total channel length (3.5 µm). A slight decrease in the ON‐current from 5.5 to 3.9 × 10^−6^ A and a negative shift of *V*_th_ from 35 to 22 V as the e‐beam dose increased were observed, indicating a gradual enhancement of the n‐doping effect. This is consistent with previous studies that proposed e‐beam irradiation causing an n‐type doping effect on the 2D materials by inducing more trapped charges in the SiO_2_ layer with the increased e‐beam dose.^[^ ^18^, ^19^, ^20^, ^21^, ^22^ ^]^

In fact, our previous study confirmed the n‐type doping effect of pristine WSe_2_ via a self‐built electrostatic potential using e‐beam irradiation.^[^ ^18^ ^]^ To confirm the contribution of the self‐built electrostatic potential in the dielectric layer, we quantitatively investigated the behavior of trapped charges at the WSe_2_/SiO_2_ interface governed by the gate bias by measuring the hysteresis in the transfer curves of the devices. At the WSe_2_/SiO_2_ interface, the existence of electron trap sites is unavoidable because of oxygen vacancies, dangling bonds and ambient adsorbates near the interface, which typically results in clockwise hysteresis in the transfer characteristics during forward and reverse bias sweeping.^[^ ^37^, ^38^, ^39^, ^40^ ^]^ Figure S11a, Supporting Information, shows the transfer curve of the pristine WSe_2_ FETs with a clockwise hysteresis loop. Figure 4c shows the effect of the interface trap sites on the transport behavior of the pristine WSe_2_ FETs based on the hysteresis behaviors. With a forward bias (negative initial gate bias), negative interface charges in the SiO_2_ can de‐trap electrons from the trap sites, leading to n‐type doping of the WSe_2_ channel. In contrast, with a backward bias (positive initial gate bias), the trap sites can be filled with electrons moving from the WSe_2_ layer, contributing to a lower n‐doped state in the WSe_2_ channel. Thus, clockwise hysteresis was observed, proving the presence of electron trap sites at the WSe_2_/SiO_2_ interface.^[^ ^37^, ^38^ ^]^

Figure S11b, Supporting Information, shows a counterclockwise hysteresis in the transfer characteristics of a p^+^–p–p^+^ seamless junction FET after oxidation and e‐beam irradiation, confirming the presence of electron trap sites in the junction FETs. However, the contribution of these electron trap sites is negligible after oxidation, as WO*_x_* strongly p‐dopes the WSe_2_ layer, resulting in a significant accumulation of hole charges even at forward bias, as illustrated in Figure 4d. More trap charges can be formed in the SiO_2_ layer by irradiating the e‐beam, forming a stronger self‐built electrostatic potential, as shown by the dose‐dependent transfer curves in Figure 4b. The self‐built potential induced by e‐beam irradiation contributes to the de‐trapping of electrons from the interface trap sites.^[^ ^18^, ^19^, ^20^, ^21^, ^22^ ^]^ Thus, the de‐trapped electrons are recombined with the hole charges in the WSe_2_ layer, as illustrated in Figure 4e. We believe that these de‐trapped electron charges cause n‐doping effects by compensating the hole charges in WSe_2_ inducing a weakly p‐doped region within p^+^‐WSe_2_ to form the lateral p^+^–p–p^+^ junction. Although a noticeable hysteresis window is observed in our p^+^–p–p^+^ junction FET, an appropriate range of applied *V*_GS_ can overcome this drawback. Figure S11b, Supporting Information, illustrates the transfer characteristics of the seamless junction FETs with various ranges of *V*_GS_ sweeping. The hysteresis windows became negligible by decreasing the *V*_GS_ range to 20 V, as the trap sites cannot effectively trap and de‐trap electrons with such small *V*_GS_ biases while maintaining the OFF state of the device. This suggests that the observed hysteresis is mainly attributed to interface trap charges rather than e‐beam‐induced charges in the SiO_2_ layer.

Notably, the ability to pattern doped regions selectively for controlling *V*_th_ precisely is extremely important in the CMOS architecture design. In conventional Si technology, ion implantation processes with different ion densities or energies are utilized to modulate the *V*_th_ of the devices, which is incompatible with the ultra‐thin body of 2D semiconductors. The *V*_th_ of 2D FETs can be precisely controlled using our technique, by simply varying the e‐beam area dose and pattern size, as shown in Figure 4b and Figure S12, Supporting Information. The *V*_th_ of the device shifted to negative voltages by increasing the e‐beam dose with a fixed irradiated area. By varying the size of the e‐beam pattern, the channel length can be changed because the e‐beam irradiated (p‐doped) region is considered as the effective channel in our device geometry (see Figure 2d). Such a change in channel length can result in *V*_th_ shift, which is a well‐known phenomenon in thin film oxide transistors. When the transistor has highly doped contact regions, the charge carrier diffusion takes place between channel and contact regions, which shifts *V*_th_ significantly.^[^ ^41^, ^42^, ^43^ ^]^

The minimum e‐beam pattern size tested in this study was ≈70 nm, as shown in Figure S12b, Supporting Information. However, with the channel length, the device did not exhibit the OFF state, as the potential barrier formed by e‐beam irradiation did not effectively block the hole carriers passing from the source to the drain owing to the short‐channel effect, for example, drain‐induced barrier lowering (DIBL).^[^ ^44^, ^45^ ^]^ Thus, further optimization is required to achieve extremely short‐channel devices smaller than 70 nm. As shown in Figure S12c,d, Supporting Information, we perform a tentative remedy to overcome the limitations of our device. By increasing the length of the contact area to >2 µm, a 70 nm e‐beam pattern can induce the OFF state for p^+^‐WSe_2_ FETs, which might be attributed to the reduced lateral electric field with a longer total device length. Das et al. suggested an effective way to overcome drain‐induced barrier lowering (DIBL) by using high‐k dielectrics^[^ ^46^ ^]^ which may also help to scale down the e‐beam pattern size <70 nm.

**Figure 5:**
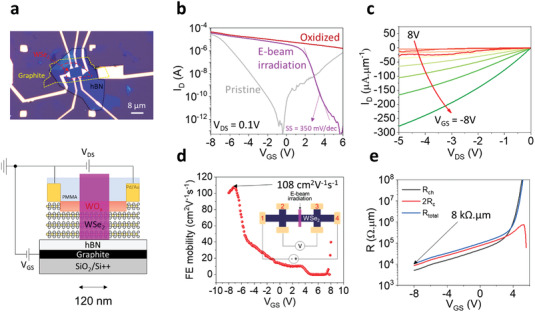
Graphite back‐gated p^+^–p–p^+^ 2D WSe_2_ FET. a) Optical micrograph and schematic of the device. b) Transfer curves measured at each fabrication step. c) Output curves after e‐beam irradiation showing high saturation current. d) Field‐effect mobility of the device after e‐beam irradiation. e) *R*_ch_ and 2*R*_C_ extracted from the four‐probe measurement.

To obtain high‐performance p‐FETs using our techniques, we fabricated graphite back‐gated devices with ≈20 nm thick hBN, as shown in **Figure** **5**a. A WSe_2_ flake was stacked on hBN/graphite to form a graphite back‐gated device prior to the same process steps used for the devices on the SiO_2_/Si substrate. Finally, a 120 nm p‐WSe_2_ channel was formed in the middle of the Hall bar device. The transfer characteristics of the device demonstrate a similar transition of electrical performance to the devices on SiO_2_ after both oxidation and e‐beam irradiation, exhibiting improved ON‐current, subthreshold swing (SS), and on/off ratio (Figure 5b). The thin hBN gate insulator contributed to the significantly improved on/off ratio (≈10^9^ with maximum and minimum currents of 3.6 × 10^–5^ and 6.7 × 10^–14^ A, respectively) after e‐beam irradiation, indicating that our technique can be universally applied to arbitrary substrates. The linear output curves reveal an extremely high saturation current (−280 μA μm^−1^ at *V*_DS_ = −5 V and *V*_GS_ = −8 V), as shown in Figure 5c. The increased ON‐current and high saturation current, which are comparable to those of state‐of‐the‐art devices (see **Table** **1**), can be attributed to the clean interface between hBN and WSe_2_ without dangling bonds. The field‐effect mobility (μ_FET_) was extracted from the four‐probe measurements with the device configuration shown in the inset of Figure 5d. Here, we considered the effective channel length to be 120 nm, because most of the voltage drop occurred across the weak p‐type region, rather than the heavily p‐doped region. The field‐effect mobility was determined to be ≈108 cm^2^ V^−1^ s^−1^. Moreover, we extracted the *R*_C_ ( = *R*_M–S_ + *R*_p+_) by four‐probe measurements showing a low *R*_C_ of ≈4 kΩ µm at *V*_GS_ = −8 V (*n* = 9 × 10^12^ cm^−2^), owing to the low impurity density and efficient gate control attributed to the ultra‐flat and thin hBN. Figure S13, Supporting Information, describes the extraction of µ_FET_ and *R*_C_. Furthermore, as shown in Figure 2d, we consider *R*_c_ as *R*_M–S_ + *R*_p+_, which includes not only MS contact resistance but also a part of p^+^‐WSe_2_ resistance. Thus, we also calculated the *R*_M–S_ of our device using the conventional four‐probe measurements for oxidized WSe_2_ to fairly compare with other reports. As shown in Figure S14b, Supporting Information, we obtained *R*_M–S_ of 2.3 kΩ µm for oxidized WSe_2_, which was comparable to other reports (≈1–3 kΩ µm).^[^ ^16^, ^23^ ^]^ The four‐probe measurements at each processing step are shown in Figure S14c, Supporting Information. The 4pp resistance (which includes *R*_channel_) was just slightly increased after e‐beam irradiation, suggesting that the contribution of defects induced by e‐beam is minor to the channel resistance. Figure S15a, Supporting Information, shows the extracted 2*R*_C_ for a graphite back gated WSe_2_ device with a 500 nm e‐beam pattern at each processing step. The 2*R*_C_ of the device showed a negligible change during the process. Thus, we believe that PMMA coating and e‐beam irradiation have a minor effect on the contact properties.

**Table 1:** Comparison of the electrical performance with WSe_2_ p‐FETs enabled by various contact strategies

Processing strategies	Polarity	Contact resistance [kΩ∙µm]	Hole mobility [cm^2^ V^−1^ s^−1^]	Saturation current [µA µm^−1^]	ON/OFF ratio
This work	p‐type	4	108 (RT)	−280 (*V*_DS_ = −5 V, *V*_GS_ = −8 V)	10^9^
Transferred via contact^[^ ^47^ ^]^	p‐type	3.5	195 (RT)	−200 (*V*_DS_ = −1.5 V, *V*_GS_ = −100 V)	10^6^
Pt pre patterning^[^ ^48^ ^]^	p‐type	100	140 (RT)	−6 (*V*_DS_ = −5 V, *V*_GS_ = −5 V)	10^6^
Au doping + ion gel^[^ ^49^ ^]^	p‐type	NA	100 (RT)	NA	10^6^
NO_2_ absorption^[^ ^50^ ^]^	p‐type	NA	250 (RT)	−10 (*V*_DS_ = −1.5 V, *V*_GS_ = −1.7 V)	>10^6^
Polymer electrolyte^[^ ^51^ ^]^	p‐type	10	180 (RT)	NA	NA
2D/2D contact (NbWSe_2_)^[^ ^52^ ^]^	p‐type	0.3	220 (RT)	−320 (*V*_DS_ = −1.5 V, *V*_GS_ = −130 V)	10^9^
Gr electrodes + ionic liquid^[^ ^53^ ^]^	p‐type	2	200 (160 K)	−180 (*V*_DS_ = −5 V, *V*_GS_ = −100 V)	10^7^
1 L WO*_x_* + 3L WSe_2_^[^ ^17^ ^]^	p‐type	66	50 (RT)	NA	10^7^
XeF_2_ thinning^[^ ^54^ ^]^	p‐type	>1000	0.35 (RT)	NA	10^4^
Al_2_O_3_/CYTOP fluoropolymer^[^ ^55^ ^]^	p‐type	NA	100 (RT)	NA	10^7^
Van der Waals contact^[^ ^30^ ^]^	p‐type	14	16 (RT)	NA	10^7^
3 L WO*_x_* + 5L WSe_2_^[^ ^23^ ^]^	p‐type	0.5	NA	−320 (*V*_DS_ = −1 V)	<10
Rapid flame synthesis MoO_3_^[^ ^56^ ^]^	p‐type	0.8	NA	1000 (*V*_DS_ = 5 V, *V*_GS_ = −15V	<10
NO absorption^[^ ^57^ ^]^	p‐type	0.95	NA	−300 (*V*_DS_ = −1 V)	>10^6^

“NA” indicates “not available in the paper”.

To confirm the low‐temperature stability, electrical measurements for the 500 nm e‐beam pattern device with graphite back‐gate were conducted at various temperatures down to 10 K, as shown in Figure S15b, Supporting Information. The device still performed well with a clear ON/OFF switching at 10 K, indicating that the e‐beam‐irradiated doped area was well preserved at such low temperatures. As shown in Figure S15c, Supporting Information, the subthreshold swing (SS) of the graphite‐gated device was further reduced from 440 to 220 mV/dec, when the temperature was lowered from 300 to 10 K, depicting thermionic transport. These results demonstrate the robustness of our technique for fabricating high‐performance short‐channel devices that can operate well at low temperatures.

## 3 Conclusion

An effective approach for patterning the doping profile of WO*_x_* via selective e‐beam patterning onto PMMA‐passivated p^+^‐WSe_2_ was demonstrated. The gate‐tunable optical responses of our seamless p^+^–p junction diode prove the effectiveness of the e‐beam irradiation for high‐performance switching devices with heavily doped TMDs. The generation of trapped charges in the dielectric layer leads to the restoration of the OFF state in p^+^‐WSe_2_, which remedies the downside of the SCTD. Furthermore, various e‐beam doses and pattern sizes written in the middle of the device allow the accurate modulation of *V*_th_. High‐performance short‐channel devices with a high μ_FET_, on/off ratio, saturation current and low *R*_C_ are observed, using hBN as the gate dielectric. The combination of SCTD and e‐beam irradiation provides efficient switching and selective patterning for seamless semiconductor junction devices.

## 4 Experimental Section

### Device Fabrication Process

2D materials (hBN and flux‐grown WSe_2_) were mechanically exfoliated by the scotch‐tape method before being transferred to a degenerately p‐doped Si wafer covered by 285 nm of thermally grown SiO_2_, which served as a global gate dielectric. The thickness of the WSe_2_ flakes (<5 nm) was determined by optical contrast and confirmed by AFM, as shown in Figure S2, Supporting Information. The PMMA A6 resist was spin‐coated to form a mask, followed by EBL for device patterning. Subsequently, the Pd/Au (5/100 nm) metal contacts were directly deposited using an e‐beam evaporator at a vacuum pressure of 5 × 10^−7^ Torr. After measuring the pristine state, the device underwent an oxygen plasma treatment at room temperature (50 sccm, 30 mTorr, 50 W, and 200 s). Subsequently, the device was passivated with either PMMA A6 or PMMA A2 with a spin‐coating speed of 4000 rpm for 90 s. For precise doping of the device, e‐beam irradiation was performed using a JEOL JSM‐7001F SEM system with a 20 keV acceleration voltage and various doses in the range from 400 to 1000 µC cm^−2^.

### Device Characterization

All electrical measurements were performed using a semiconductor parameter analyzer (Agilent 4155C) connected to a vacuum probe station with pressure maintained at 20 mTorr. Low‐temperature measurements were performed using a Janis cryostat with a source meter (Keithley 2636 B). Raman and PL measurements were performed at room temperature using a multifunctional microscope (UniRam) with a laser excitation wavelength of 532 nm. The size of the pattern and the thickness of the flakes were measured using the non‐contact mode AFM (Park systems) under ambient conditions.

## Conflict of Interest

The authors declare no conflict of interest.

## Author Contributions

T.D.N. and M.S.C. contributed equally to this work. T.D.N. and M.S.C. conceived the project and performed experiments. W.J.Y. supervised the project and guided the experiment. T.D.N. and M.S.C. fabricated the devices with the contributions of M.L. and F.A. for e‐beam lithography. Y.H., N.A., and C.L. contributed to low‐temperature measurements. S.L. and J.H. provided the flux‐grown WSe_2_ crystals. T.D.N. and M.S.C. wrote the manuscript under the supervision of W.J.Y. All authors reviewed and commented on the manuscript.

## Supporting information

Supporting information

## Data Availability

The data that support the findings of this study are available from the corresponding author upon reasonable request.
