# Supplementary material for: Selective Electron Beam Patterning of Oxygen‐Doped WSe2 for Seamless Lateral Junction Transistors
Source: Adv Sci (Weinh). 2022 Jul 19;9(26):2202465. doi: 10.1002/advs.202202465 (PMC9475546; doi:10.1002/advs.202202465)
Supplement: Supplementary file 1 — Supporting information [file ADVS-9-2202465-s001.pdf]

ADVANCED SCIENCE
Open Access

# Supporting Information

for *Adv. Sci.*, DOI 10.1002/advs.202202465

Selective Electron Beam Patterning of Oxygen-Doped $WSe_2$ for Seamless Lateral Junction Transistors

*Tien Dat Ngo, Min Sup Choi, Myeongjin Lee, Fida Ali, Yasir Hassan, Nasir Ali, Song Liu, Changgu Lee, James Hone and Won Jong Yoo**

# Supporting Information

## Selective electron beam patterning of oxygen doped $WSe_2$ for seamless lateral junction transistors

Tien Dat Ngo[‡a], Min Sup Choi[‡a], Myeongjin Lee[a], Fida Ali[a], Yasir Hassan[a], Nasir Ali[a], Song Liu[b], Changgu Lee[a,c], James Hone[b] and Won Jong Yoo[*a]

[a]SKKU Advanced Institute of Nano Technology, Sungkyunkwan University, Suwon, Gyeonggi-do 16419, Korea

[b]Department of Mechanical Engineering, Columbia University, New York, NY, USA

[c]School of Mechanical Engineering, Sungkyunkwan University, Suwon, South Korea

[‡] These authors contributed equally to this work.

[*] e-mail: yoowj@skku.edu

*Keywords: 2D semiconductors, oxygen plasma, e-beam irradiation, tungsten oxide, patterning doping profiles.*

# Oxidized $WSe_2$ seamless junction patterned by e-beam irradiation

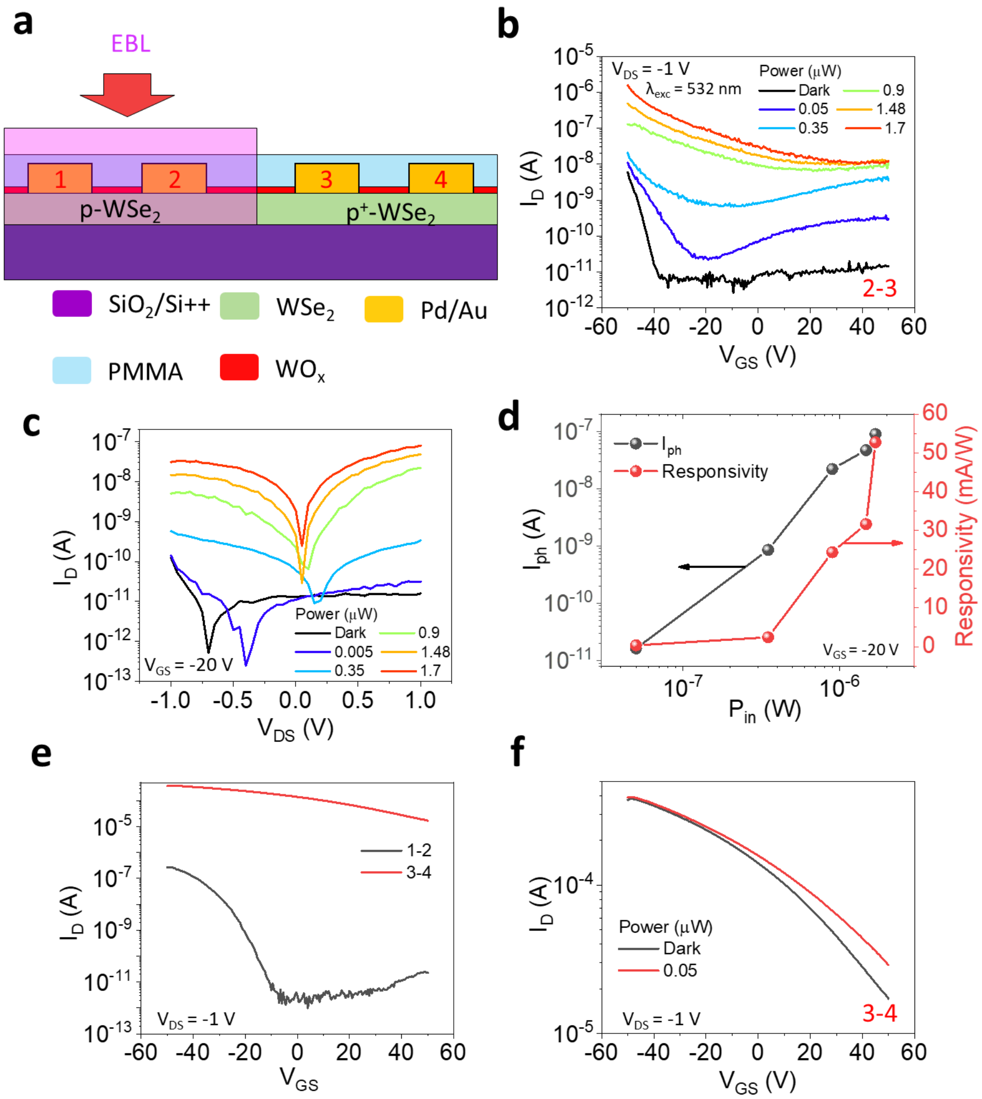


**Figure S1.** a) Device schematic of oxidized $WSe_2$ p-p$^+$ seamless junction patterned by e-beam irradiation. b) Transfer and c) output characteristics of the device measured under dark and 532 nm laser illumination condition. d) Photocurrent and responsivity of the device at $V_{GS}$ = -20 V. e) Transfer characteristics of p$^+$-$WSe_2$ (measured between electrodes 3 and 4) and e-beam irradiated p-$WSe_2$ (measured between electrodes 1 and 2). f) Transfer characteristics of the p$^+$-$WSe_2$ (measured between electrodes 3 and 4) device measured under dark and 532 nm laser illumination condition.

Formula used to extract the position of Fermi level of each side in our p-p$^+$ junction diode shown in Figure 1d-f is expressed by below equation,

$$n = n_i e^{(E_F - E_i)/kT}, \qquad (1)$$

where $n$ is the carrier density of the device, $n_i$ is the intrinsic carrier density of $WSe_2$ (1.8 × $10^4$ $cm^{-2}$),[1] $kT$ is the thermal energy, and $E_F(E_i)$ is the Fermi level (intrinsic Fermi level, 4.52 eV),[2] respectively. The $n$ is extracted by the equation, $n = \sigma/e\mu_{FET}$, where $\sigma$ is the conductance, $e$ is the electron charge, $\mu_{FET}$ is the field effect mobility determined by the following equation:

$$\mu_{FET} = (1/C_{ox})|d\sigma/dV_{GS}|, \qquad (2)$$

where $C_{ox}$ is the oxide capacitance (1.2 × $10^{-8}$ F/$cm^2$), $d\sigma/dV_{GS}$ is the slope of transfer characteristics from Figure S1e. The calculated parameters used to extract the position of Fermi level of p$^+$- and p-type $WSe_2$ are shown in Table S1.

**Table S1**. Extracted parameters of p-p$^+$ oxidized $WSe_2$ seamless junction.

| $V_{GS}$ (V) | Carrier type | $\sigma$ (S) | $\mu_{FET}$ ($cm^2V^{-1}s^{-1}$) | $n$ ($cm^{-2}$) | Work function (eV) | Built-in potential (eV) |
|---|---|---|---|---|---|---|
| -50 | p$^+$ | 3.74e-5 | 33 | 7.02e12 | 5.03 | 0.05 |
| | p | 2.66e-8 | 0.138 | 1.2e12 | 4.98 | |
| -20 | p$^+$ | 2.36e-5 | 33 | 4.42e12 | 5.02 | 0.18 |
| | p | 8.17e-11 | 0.138 | 3.69e9 | 4.84 | |

## Atomic force microscopy (AFM) images of $WSe_2$ flake and extraction of heights and lengths of patterned PMMA layer

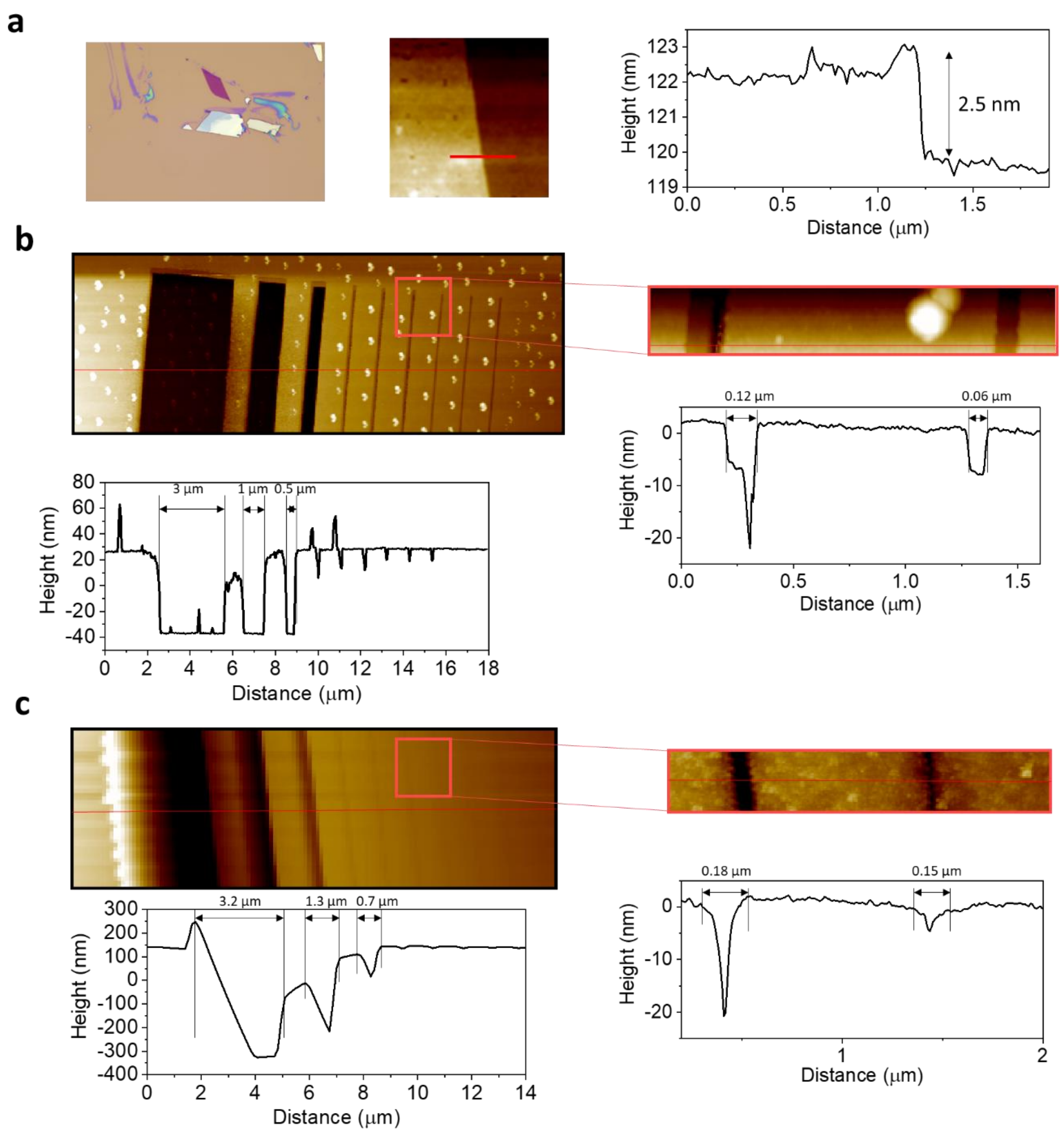


**Figure S2.** a) Optical and AFM images with height profile of a representative flake (~2.5 nm) used in this work. Extraction of actual heights and lengths from array of e-beam patterns for b) PMMA A2 and c) PMMA A6.

Figure S2a shows the AFM image of a representative $WSe_2$ flake used in this study. The thickness of the flake less than 5 nm (~ 2.5nm) was confirmed. All other flakes were also identified by optical contrast and AFM images. Figure S2b,c represent actual measured heights and lengths of the e-beam patterns written on PMMA A2 and A6, respectively. The arrays of pattern with the lengths of 3000 nm, 1000 nm, 500 nm, 100 nm $\times$ 3, 50 nm $\times$ 3 were patterned by e-beam lithography, followed by a development process with a developer (IPA : Water = 3 : 1). The actual sizes of the e-beam pattern were extracted from line profiles of AFM images. With PMMA A2, 3000 nm, 1000 nm, 500 nm patterns showed exactly the same size with CAD patterns. However, smaller patterns with 100 nm and 50 nm lengths showed 120 nm and 70 nm lengths, respectively, which are slightly larger than our intended design. For PMMA A6, however, the actual size of patterns was extracted as 3200 nm, 1300 nm, 700 nm, 180 nm, 150 nm for the same designed lengths, which is much far from the intended size of pattern. The significant discrepancy of lengths between original CAD patterns and actual patterns was attributed to enhanced electron scattering in the thick PMMA layer as discussed in the Monte Carlo simulation. In this work, we consider the actual size of patterns based on our AFM data rather than original CAD patterns. The thickness of PMMA A2 and A6 were around 80 and 380 nm, respectively. All the parameters discussed above are summarized in Table S2.

**Table S2**. Parameters of pattern written by EBL on PMMA with different thicknesses.

| | Thickness (nm) | **Intended length of pattern (nm)** | | | | |
|---|---|---|---|---|---|---|
| | | 50 | 100 | 500 | 1000 | 3000 |
| | | **Actual length of pattern (nm)** | | | | |
| PMMA A2 | 80 | 70 | 120 | 500 | 1000 | 3000 |
| PMMA A6 | 380 | 150 | 180 | 700 | 1300 | 3200 |

## Electrical measurements at each processing step

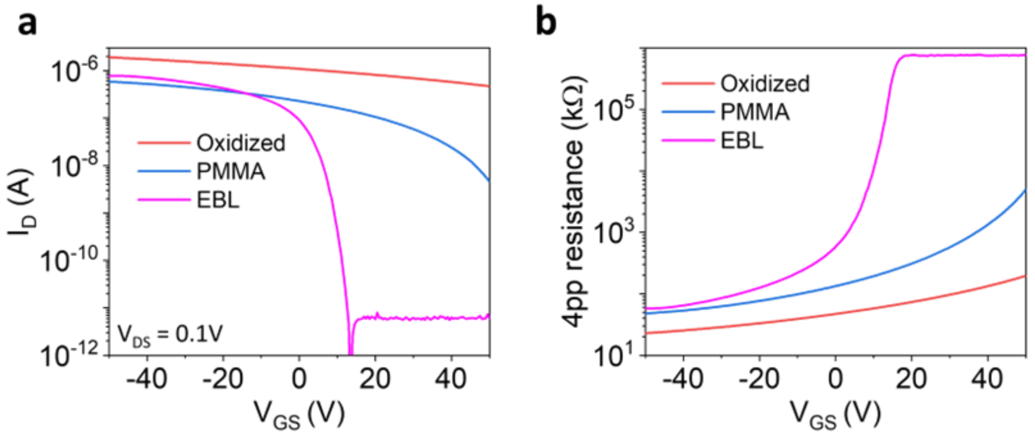


**Figure S3**. (a) Transfer curves and (b) $R_{4pp}$ of $WSe_2$ FETs at oxidized, PMMA coated, and e-beam irradiated states.

## Effect of e-beam irradiation on channel area of $p^+$-$WSe_2$ FETs

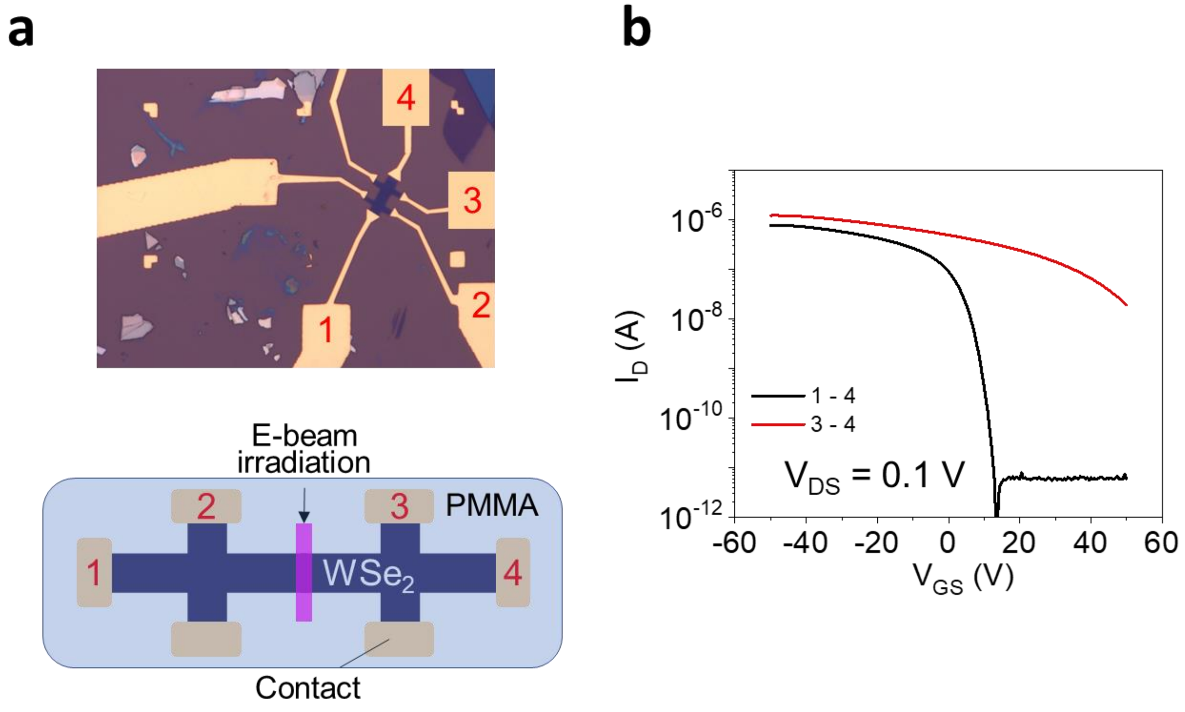


**Figure S4**. a) Optical image and schematic of the device to investigate the effect of e-beam irradiation on channel area. b) Transfer curves of the devices with e-beam irradiation (measured

between electrodes 1 and 4) and without e-beam irradiation (measured between electrodes 3 and 4). E-beam irradiation just locally affected the density of exposed area while the nearby unexposed area remained $p^+$-$WSe_2$ with low ON/OFF ratio.

## Time stability of e-beam irradiated $p^+$-$WSe_2$ FETs

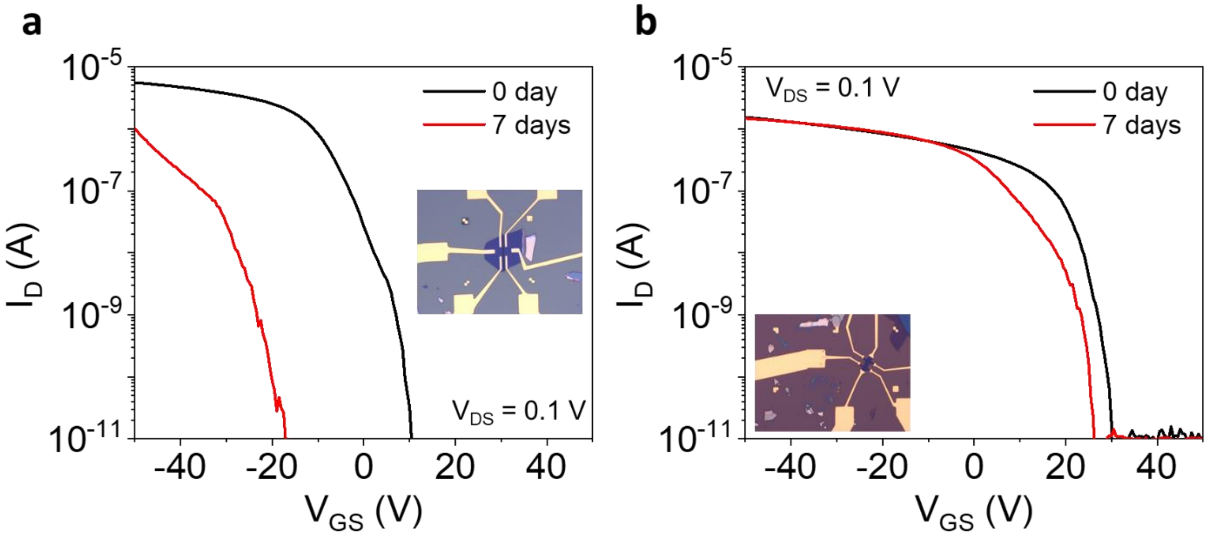


**Figure S5**. a) Transfer curves of e-beam irradiated $p^+$-$WSe_2$ FETs a) without PMMA and b) with PMMA as-fabricated (0 day) and after 7 days.

## Output curves of the device during fabrication process

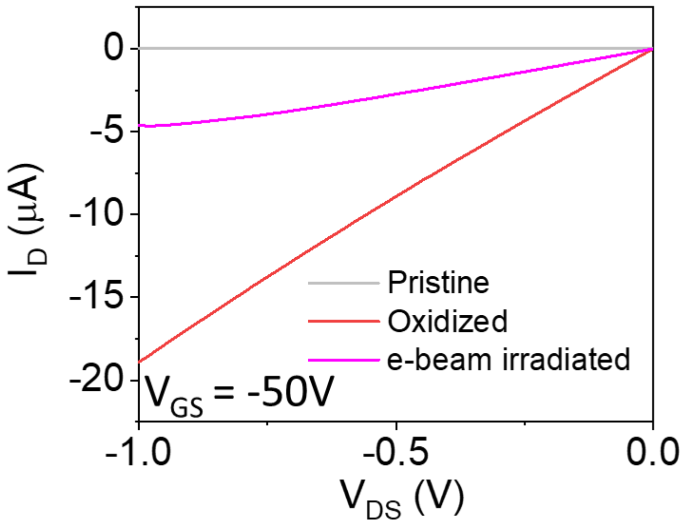


**Figure S6**. Output curves of the device at each fabrication step; pristine, oxidized, and e-beam irradiated states.

# Effect of PMMA thickness on electrical performances

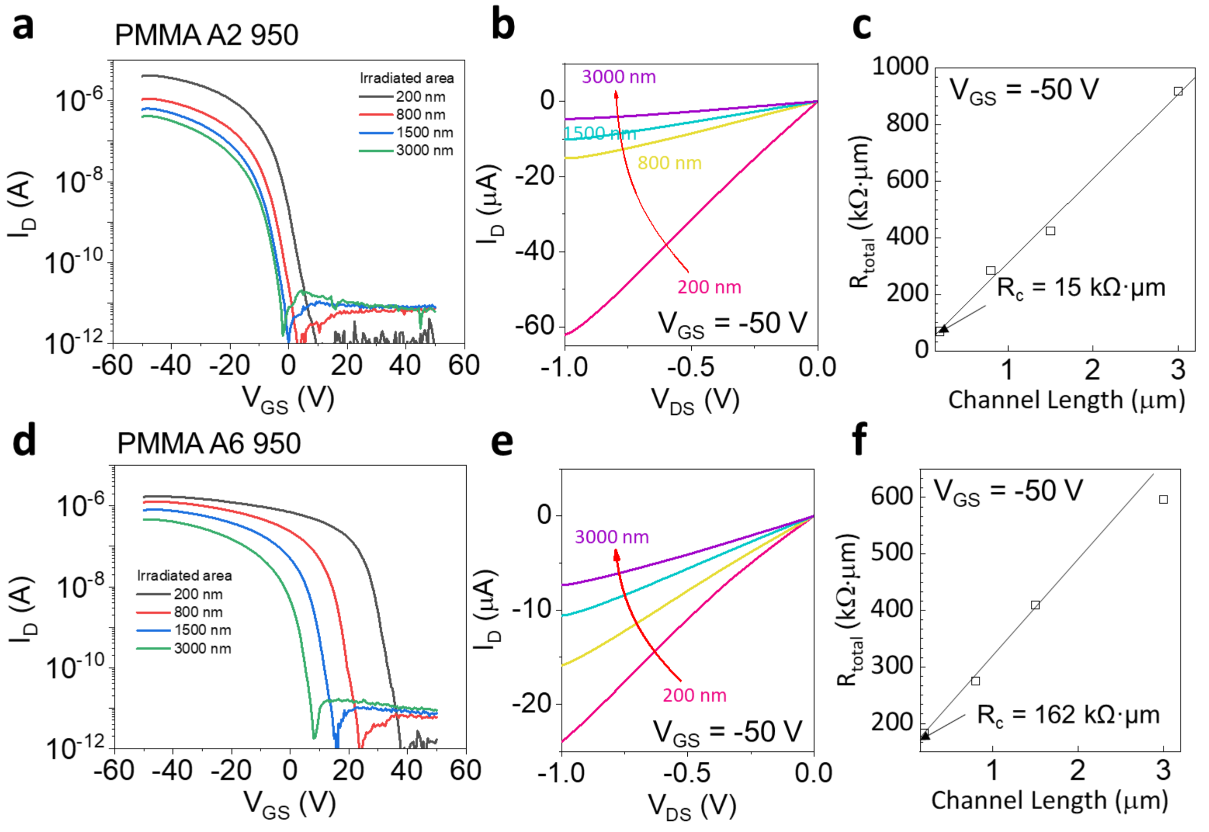


**Figure S7**. a) Transfer curves, b) Output curves, and c) TLM fitting of the device passivated by PMMA 950 A2 layer. d) Transfer curves, e) Output curves, and f) TLM fitting of the device passivated by PMMA 950 A6 layer.

# Monte Carlo simulation of e-beam lithographic patterns for PMMA A2 and A6

To investigate the impact of PMMA thickness on accuracy of our e-beam lithographic patterns, we carried out a Monte Carlo simulation using CASINO.[3] We set the thicknesses of PMMA A2 and A6 as 80 and 350 nm, respectively, that is consistent with Figure S1. In the simulation, thickness of $WO_x$, $WSe_2$, $SiO_2$ are 1, 2.5, 285 nm, respectively. We used the same density of PMMA for both A2 and A6 in this simulation as their molecular weight (950,000 g/mol) are same. As shown in Figure S8, the actual lengths of A2 have a slight difference (~20 nm) when the patterned lengths were less than 100 nm. However, the A6 showed a significant difference between intended length and actual length due to increased electron scattering. The obtained lengths from the simulation are well matched with the extracted lengths from AFM line profiles as listed in Table S2.

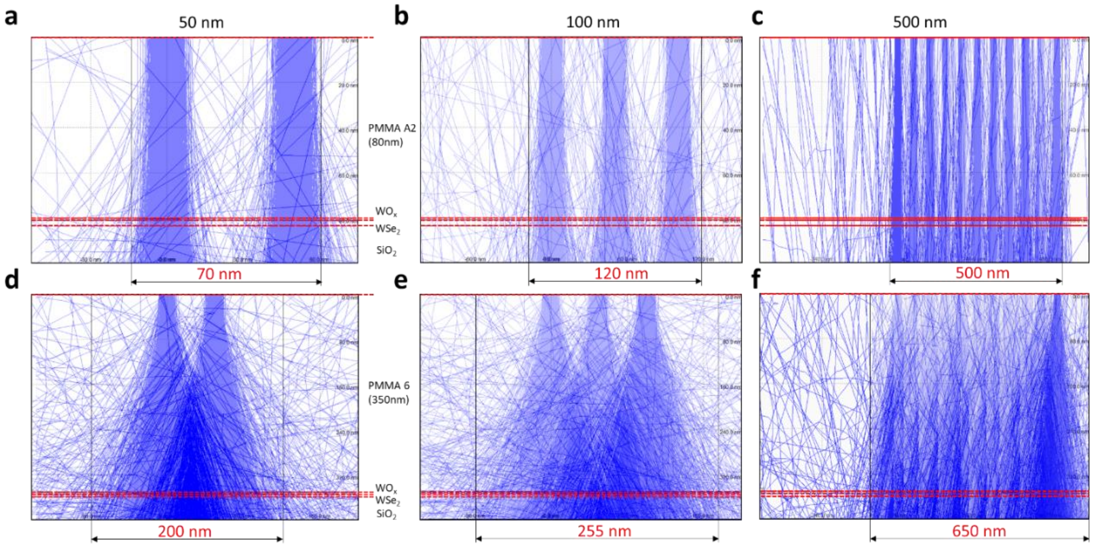


**Figure S8**. Monte Carlo simulation of spatial distribution of electrons exposed on PMMA A2 (thickness of PMMA A2 is 80 nm) with size of a) 50 nm, b) 100 nm, c) 500 nm, respectively, and PMMA A6 (thickness of PMMA A6 is 360 nm) with size of d) 50 nm, e) 100 nm, f) 500 nm, respectively.

# Photoluminescence (PL) spectra of oxidized $WSe_2$

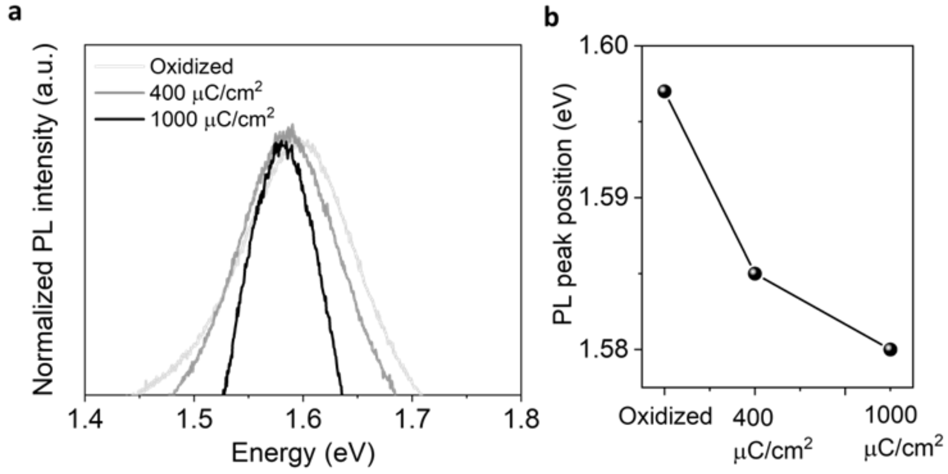


**Figure S9.** a) PL spectra and b) PL peak position of the oxidized $WSe_2$ before and after e-beam irradiation with different beam dose.

## PL spectra of $WSe_2$ during fabrication process

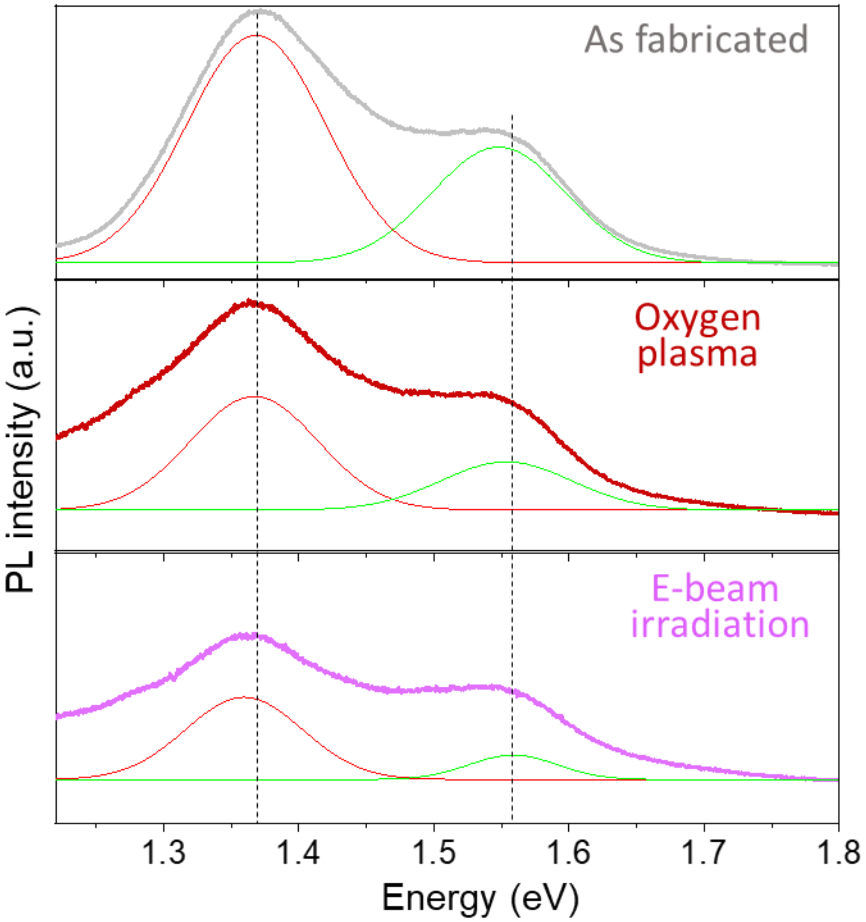


**Figure S10.** PL spectra of $WSe_2$ for fabricated (grey), oxygen plasma treated (dark red), e-beam irradiated (pink) states. Gaussian fits were used to deconvolute the doublet curve into two distinct exciton peaks (red and green curves) from direct and indirect bandgaps.

## Hysteresis behavior of pristine and seamless p$^+$-p-p$^+$ $WSe_2$ device

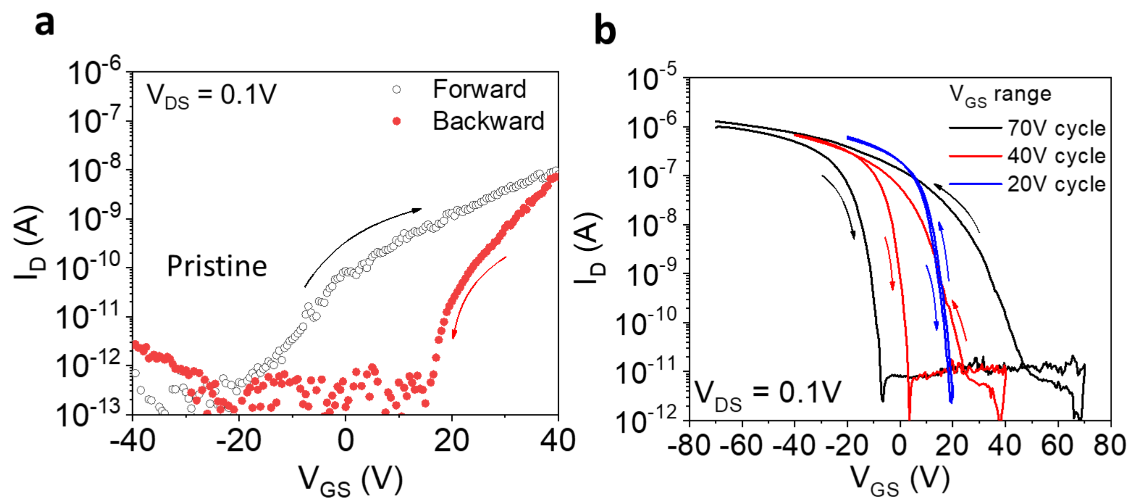


**Figure S11.** a) Transfer characteristics of pristine $WSe_2$ FETs with hysteresis window. b) Transfer characteristics of seamless p$^+$-p-p$^+$ homogeneous junction FETs maintaining OFF state and improved hysteresis window by reducing the $V_{GS}$ range.

## E-beam irradiated length dependent threshold voltage, and the effect of total device length on electrical performances

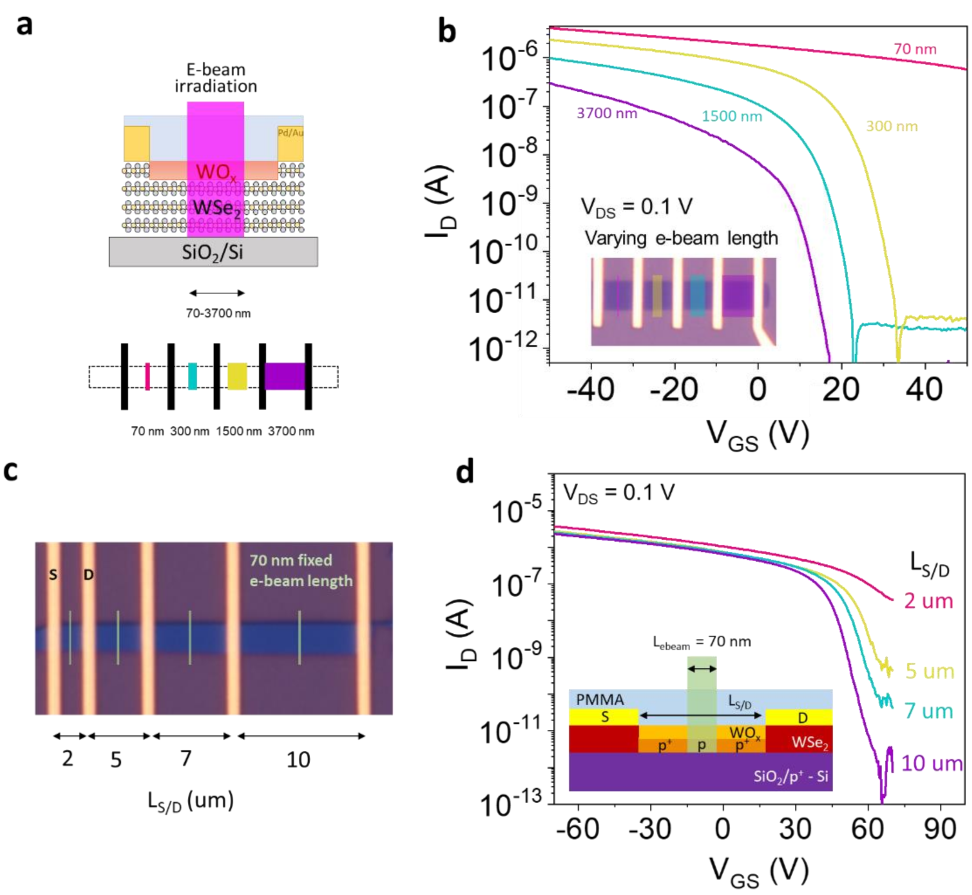


**Figure S12.** a) Schematic to illustrate the device geometries and irradiated e-beam lengths. b) Transfer curves with various e-beam irradiated lengths (70 ~ 3700 nm). The inset is optical microscopic image of the device. c) Optical image and d) transfer characteristics of the 70 nm e-beam irradiated $p^+$-$WSe_2$ FETs with various total device lengths ($L_{S/D}$).

## Extraction of contact resistance and mobility from four-point probe (4pp) measurements

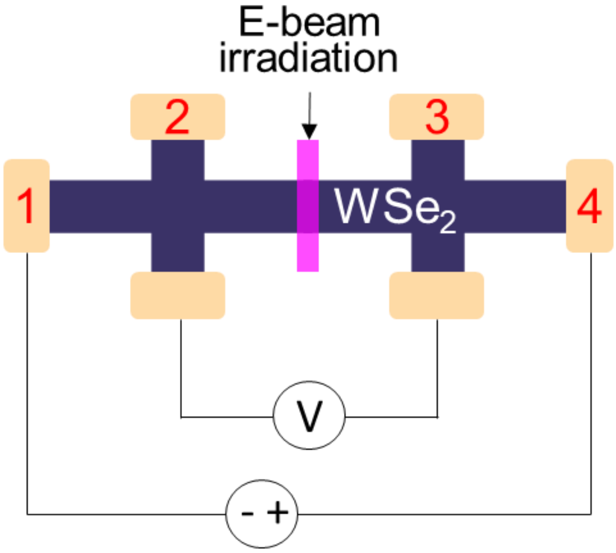


**Figure S13.** Device schematic used for 4pp measurements.

In 2pp measurement, a fixed voltage ($V_{DS}$) is applied across the contacts 1 and 4, and the $I_D$ is measured across the same contact to extract 2pp resistance, $R_{2pp}=W\cdot(V_{DS}/I_D)$. In 4pp measurement, a voltage drop ($V_{23}$) across the channel can be measured between contacts 2 and 3 to exclude the effect of contact resistance. The extracted 4pp resistance is expressed by $R_{4pp}=W\cdot(V_{23}/I_D)$. The $R_{4pp}$ is mainly affected by e-beam irradiated area as the resistance of contact part is not affected by e-beam irradiation as discussed in Figure S3. Thus, we used the device configuration as shown in Figure S13 to calculate the contact resistance expressed by $2R_C = R_{2pp} - R_{4pp}$. From the equation, we obtain 4pp mobility as follows:

$$\mu_{4pp} = \frac{1}{C_{hBN}} \cdot \frac{L_{irradiated}}{W} \cdot \frac{dG}{dV_{GS}}. \qquad (3)$$

Here, $L_{irradiated}$ and $W$ are the e-beam irradiated length and width (120 nm and 3.5 μm, respectively), $\frac{dG}{dV_{GS}}$ is the transconductance of the device, and $C_{hBN}$ is the capacitance of 20 nm thick hBN (~120 nF/cm$^2$).[4]

## Electrical measurements of graphite gated device

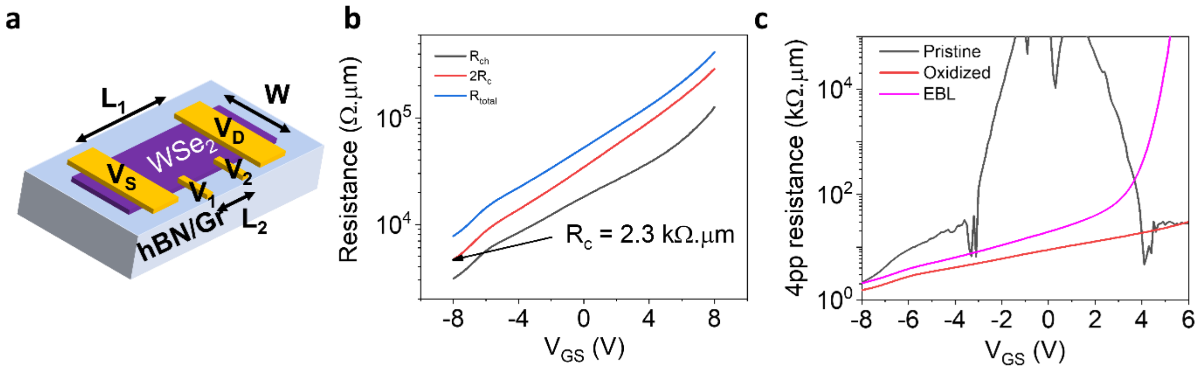


**Figure S14.** a) Device configuration for 4-probe measurements of oxidized $WSe_2$. b) $R_{M\text{-}S}$ of the oxidized $WSe_2$ device before e-beam irradiation. c) 4pp resistance of the graphite gated device at each processing step.

Here, we recalculated the $R_{M\text{-}S}$ of our device using the conventional 4-probe measurements for oxidized $WSe_2$ to fairly compare with other reports. In Figure S14b, we extracted the $2R_{M\text{-}S} = R_{2pp} - (L_1/L_2)R_{4pp}$, where $L_1$ = 9.2 μm, $L_2$ = 4.5 μm, $W$ = 3.5 μm, $R_{2pp} = W*V_{DS}/I_D$, and $R_{4pp} = W*(V_1\text{-}V_2)/I_D)$ by using typical 4-probe device configuration. From these equations, we obtained $R_{M\text{-}S}$ of 2.3 kΩ·μm for oxidized $WSe_2$, which was comparable to other reports. In Figure S14c, we showed 4pp resistance as a function of $V_{GS}$ of the $WSe_2$ device at each processing step.

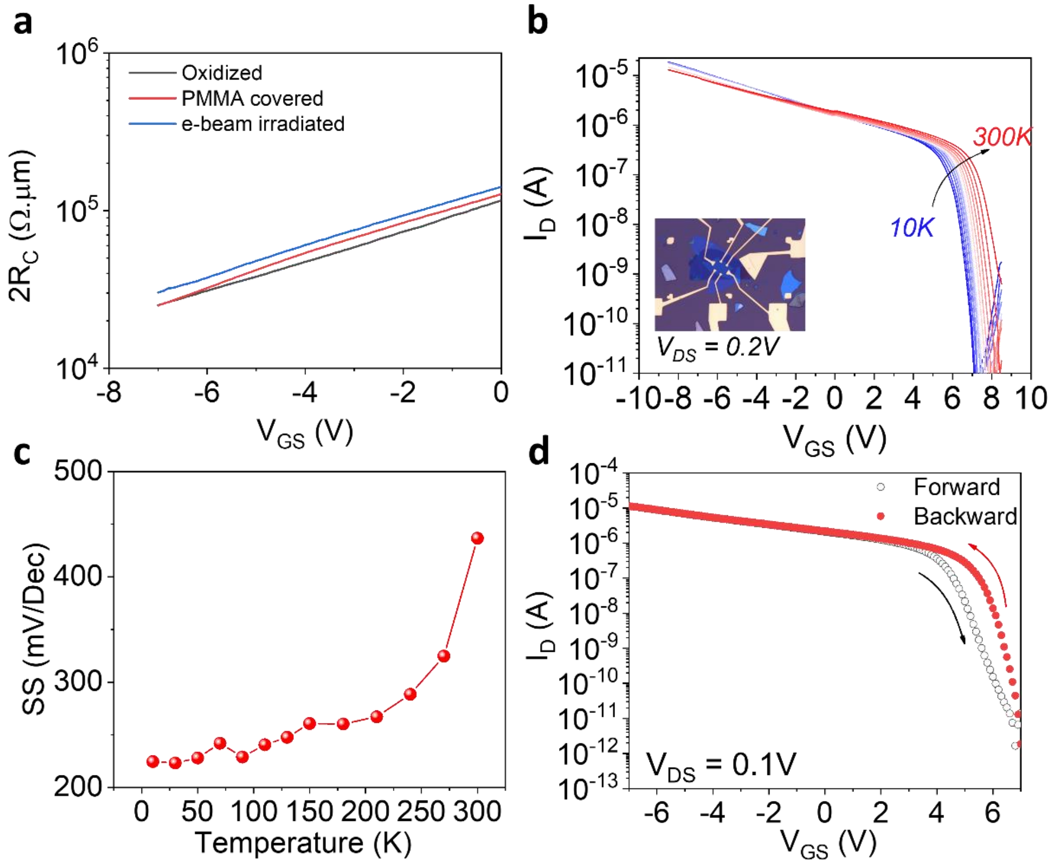


**Figure S15.** a) Contact resistance of the graphite gated device during processing steps. b) Temperature dependent transfer characteristics of the graphite gated device. The inset is optical image of the device. c) Temperature dependent subthreshold swing (SS) extracted from transfer characteristics of the graphite gated device. d) Hysteresis behavior of the graphite gated device.

# Reference


1. P. Dhakras, P. Agnihotri, J. U. Lee, *Nanotechnology* **2017**, 28, 265203.
2. J. Kang, W. Liu, D. Sarkar, D. Jena, K. Banerjee, *Phys. Rev. X* **2014**, 4, 031005.
3. D. Drouin, A. R. Couture, D. Joly, X. Tastet, V. Aimez, R. Gauvin, *Scanning* **2007**, 29, 92.
4. H. C. Movva, A. Rai, S. Kang, K. Kim, B. Fallahazad, T. Taniguchi, K. Watanabe, E. Tutuc, S. K. Banerjee, *ACS Nano* **2015**, 10, 10402.
